# *Paramirandinaguttulata* (Microthyriaceae, Microthyriales), a new lignicolous freshwater fungi from a plateau lake in Yunnan Province, China

**DOI:** 10.3897/BDJ.12.e137989

**Published:** 2024-11-22

**Authors:** Hong-Wei Shen, Dan-Feng Bao, Na Wu, Fatimah Al-Otibi, Zong-Long Luo, Kevin D. Hyde

**Affiliations:** 1 College of Agriculture and Biological Science, Dali University, Dali, China College of Agriculture and Biological Science, Dali University Dali China; 2 School of Science, Mae Fah Luang University, Chiang Rai, Thailand School of Science, Mae Fah Luang University Chiang Rai Thailand; 3 Center of Excellence in Fungal Research, Mae Fah Luang University, Chiang Rai, Thailand Center of Excellence in Fungal Research, Mae Fah Luang University Chiang Rai Thailand; 4 Engineering and Research Center for Southwest Biopharmaceutical Resource of National Education Ministry of China, Guizhou University, Guiyang, China Engineering and Research Center for Southwest Biopharmaceutical Resource of National Education Ministry of China, Guizhou University Guiyang China; 5 School of Life Science and Technology, Center for Informational Biology, University of Electronic Science and Technology of China, Chengdu, China School of Life Science and Technology, Center for Informational Biology, University of Electronic Science and Technology of China Chengdu China; 6 Department of Botany and Microbiology, College of Science, King Saud University, Riyadh, Saudi Arabia Department of Botany and Microbiology, College of Science, King Saud University Riyadh Saudi Arabia

**Keywords:** new species, Dothideomycetes, hyphomycetes, phylogeny, taxonomy

## Abstract

**Background:**

Based on unique morphological features and phylogenetic analysis of combined LSU and ITS sequences, *Paramirandinaguttulata* is established as the third species in *Paramirandina*, along with *P.aquatica* and *P.cymbiformis*. All three species of *Paramirandina* were reported from freshwater habitats in China.

**New information:**

*Paramirandinaguttulata* sp. nov. is a morphologically conspicuous hyphomycetes species, collected from a plateau lake (Dujuanhu Lake) in Yunnan Province, China. The key distinguishing characteristics of *P.guttulata* are scattered or gregarious conidiophores, polyblastic, terminal, sympodial conidiogenous cells and fusiform, cymbiform, 4–6-septate, guttulate, hyaline conidia that are solitary or in chains and with truncate base and obtuse apex. Detailed descriptions and illustrations are provided for the new species.

## Introduction

Lignicolous freshwater fungi grow on submerged woody debris in freshwater habitats and play an important role in the material and energy cycle of freshwater ecosystems (Hyde et al. 2016, Calabon et al. 2023). They have the ability to degrade lignocellulose in the wood under submerged conditions (Bucher et al. 2004). Lignicolous freshwater fungi are a highly diverse group, primarily belonging to Dothideomycetes and Sordariomycetes in Ascomycota (Hyde et al. 2016, Luo et al. 2019, Dong et al. 2020, Calabon et al. 2022, Shen et al. 2022a, Yang et al. 2023). Yunnan Province is one of the hotspots for lignicolous freshwater fungi research, a substantial number of lignicolous freshwater fungal species having been documented, predominantly from lotic freshwater habitats such as: streams and rivers (Su et al. 2016, Luo et al. 2016, Luo et al. 2017, Bao et al. 2018, Luo et al. 2018a, Luo et al. 2018b, Luo et al. 2019, Bao et al. 2020, Dong et al. 2020, Bao et al. 2021, Dong et al. 2021, Bao et al. 2023) and several studies have also focused on lentic freshwater habitats such as plateau lakes and ponds (Cai et al. 2002, Luo et al. 2004, Huang et al. 2022, Li et al. 2022, Shen et al. 2022b, Luan et al. 2023, Shen et al. 2023, Li et al. 2024). We are currently investigating the diversity of lignicolous freshwater fungi from plateau lakes in Yunnan Province, where several novel and intriguing species have been described and illustrated (Huang et al. 2022, Li et al. 2022, Shen et al. 2022b, Luan et al. 2023, Shen et al. 2023, Li et al. 2024).

Microthyriaceae was introduced by Saccardo (1883) with *Microthyrium* as the type genus. Based on morphological examination of the generic type species, Wu et al. (2010), Wu et al. (2011a), Wu et al. (2011b) and Wu et al. (2014) conducted several re-appraisals of Microthyriaceae, excluded several genera and accepted seven genera in Microthyriaceae. Wijayawardene et al. (2018) accepted nine genera in this family and later 11 genera were accepted by Hongsanan et al. (2020). Subsequently, several new genera were successively introduced into Microthyriaceae based on morphology and phylogeny (Qiao et al. 2021, Zheng et al. 2022, Liu et al. 2023). Currently, 18 genera are accepted in Microthyriaceae, including ten asexual genera (Liu et al. 2023).

*Paramirandina* was introduced by Liu et al. (2023) to accommodate two lignicolous freshwater fungi from lotic habitats in Guizhou Province, with *P.aquatica* as the type species. *Paramirandina* is morphologically similar to *Heliocephala* and *Mirandina*, but can be distinguished from them by the morphology of conidia and conidiogenous cells and also differences in phylogenetic placement (Liu et al. 2023). *Paramirandina* is characterised by macronematous, mononematous, unbranched, erect, cylindrical, septate conidiophores that are dark brown, becoming pale brown to subhyaline towards the apex; polyblastic, cylindrical to lageniform, pale brown to subhyaline, sometimes elongating percurrently conidiogenous cells; solitary or gathered in chains, fusiform, cymbiform or narrowly lunate, hyaline, septate conidia (Liu et al. 2023). The sexual morphs of *Paramirandina* have not yet been discovered and two asexual species, *P.aquatica* and *P.cymbiformis*, are currently reported from lotic freshwater habitats in Giuzhou Province, China (Liu et al. 2023).

During the investigation of lignicolous freshwater fungi from a plateau lake in Yunnan Province, a conspicuous hyphomycetes was discovered on the submerged woody substrate. A detailed morphological description and comprehensive phylogenetic analysis confirmed the distinctiveness and phylogenetic placement of the species within *Paramirandina*.

## Materials and methods

### Sample collection, specimen examination and isolation

Fresh specimens were collected from Dujuanhu Lake in Yunnan Province, China on 24 February 2023. Sample collection, processing and cultivation were performed according to Shen et al. (2023). Macromorphological characters of samples were observed using an Optec SZ 760 compound stereomicroscope. The temporarily prepared microscope slide was placed under a Nikon ECLIPSE Ni-U compound stereomicroscope for observation and microscopic morphological photography. The morphology of colonies on native substrates was photographed with a Nikon SMZ1000 stereo zoom microscope. The measurements of photomicrographs were obtained using Tarosoft (R) Image Frame Work version 0.9.7. Images were edited with Adobe Photoshop CS5 Extended v. 12.0.0.0 (Adobe Systems, San Jose, California).

Single spore isolations were performed as follows: the tip of a sterile toothpick dipped in sterile water was used to capture the conidia of the target colony directly from the specimen; the conidia were then streaked on the surface of water agar (WA) or potato dextrose agar (PDA) and incubated at room temperature overnight. The single germinated conidia were transferred to fresh PDA medium and incubated at room temperature. A few of the remaining germinated spores in the media plate were separated along with agar using a needle and transferred on to water-mounted glass slides for photographs to capture the germination position of the germ tubes. After finalising the observation and isolation, the specimens were dried under natural light, wrapped in absorbent paper and placed in a ziplock bag with mothballs. Specimens were deposited in the Herbarium of Kunming Institute of Botany, Academia Sinica (KUN-HKAS). The living cultures were deposited in Dali University Culture Collection (DLUCC). Faces of Fungi number were acquired as guidelines by Jayasiri et al. (2015) and Fungal Names were registered in Fungal Names data repository (https://nmdc.cn/fungalnames/registe).

### DNA extraction, PCR amplification and sequencing

DNA extraction, PCR amplification, sequencing and phylogenetic analysis were undertaken following the methods of Dissanayake et al. (2020). Mycelia for DNA extraction from each isolate was grown on PDA for 3–4 weeks at room temperature. Total genomic DNA was extracted from 100–300 mg axenic mycelium via scraping from the edges of the growing culture using a sterile scalpel and transferred to a 1.5 ml microcentrifuge tube using sterilised inoculum needles. The mycelium was ground to a fine powder with liquid nitrogen or quartz sand to break the cells for DNA extraction. DNA was extracted with the Trelief^TM^ Plant Genomic DNA Kit (TSP101) following the manufacturer’s guidelines.

ITS and LSU genes were amplified using ITS5/ITS4 (White et al. 1990) and LR0R/LR7 (Vilgalys and Hester 1990) primer pairs, respectively. The PCR mixture contained 12.5 μl of 2 × GS Taq PCR MasterMix (mixture of DNA Polymerase, dNTPs, Mg^2+^ and optimised buffer; Genesand Biotech, Beijing, China), 1 μl of each primer including forward primer and reverse primer (10 μM), 1 μl template DNA extract and 9.5 μl double-distilled water. The PCR thermal cycling conditions were performed following Shen et al. (2023). PCR products were purified using minicolumns, purification resin and buffer according to the manufacturer’s protocols. The PCR sequences were carried out at Beijing Tsingke Biological Engineering Technology and Services Co., Ltd (Beijing, P.R. China).

### Phylogenetic analysis

BLAST searches were performed to retrieve similar sequences from GenBank (http://www.ncbi.nlm.nih.gov, accessed on 14 Jun 2024). The sequences were aligned using MAFFT online service: multiple alignment programme MAFFT v.7 (http://mafft.cbrc.jp/alignment/server/index.html, accessed on 14 Jun 2024; Kuraku et al. (2013), Katoh et al. (2019)) and sequence trimming was performed with trimAl v.1.2 with default parameters (http://trimal.cgenomics.org for specific operation steps; Capella-Gutierrez et al. (2009)). The sequence dataset was combined using SquenceMatrix v.1.7.8 (Vaidya et al. 2011). FASTA alignment formats were changed to PHYLIP and NEXUS formats by the website: ALignment Transformation EnviRonment (ALTER) (http://sing.ei.uvigo.es/ALTER/, accessed on 14 Jun 2024).

Maximum Likelihood (ML) analysis was performed setting RAxML-HPC2 on XSEDE (8.2.12) in CIPRES Science Gateway (http://www.phylo.org/portal2; accessed on 25 Jun 2024; Stamatakis (2006), Stamatakis et al. (2008), Miller et al. (2010)), using the GTR+GAMMA model with 1000 bootstrap repetitions. Bayesian analysis was performed in MrBayes 3.2.6 (Ronquist et al. 2012) and the best-fit model of sequences evolution was estimated via MrModelTest 2.2 (Guindon and Gascuel 2003, Darriba et al. 2012). The Markov Chain Monte Carlo (MCMC) sampling approach was used to calculate posterior probabilities (PP) (Rannala and Yang 1996). Bayesian analysis of six simultaneous Markov chains were run for 1,000,000 generations and trees were sampled every thousand generation.

Phylogenetic trees were visualized using FigTree v.1.4.0 (http://tree.bio.ed.ac.uk /software/figtree/), with editing and typesetting using Adobe Illustrator (AI) (Adobe Systems Inc., San Jose, CA, USA). The new sequences were submitted in GenBank and the strain information used in this paper are provided in Table 1.

## Taxon treatments

### Paramirandina guttulata

H.W. Shen, K.D. Hyde & Z.L. Luo sp. nov.

D585C72C-D9F6-5817-B666-5AAF1FCFCAF7

https://nmdc.cn/fungalnames/:registration identifier: 572010

#### Materials

- **Type status:** Holotype. **Occurrence:** catalogNumber: KUN-HKAS 131771; occurrenceRemarks: on unknown decaying plant branch submerged in a lake; recordNumber: L2204; recordedBy: H. W. Shen; sex: anamorph; lifeStage: asexual; occurrenceID: 605EBD3A-853F-50BB-8A11-8FA4C5C39F82; **Taxon:** kingdom: Fungi; phylum: Ascomycota; class: Dothideomycetes; order: Microthyriales; family: Microthyriaceae; genus: Paramirandina; specificEpithet: guttulata; taxonRank: species; verbatimTaxonRank: sp.; nomenclaturalCode: ICN; **Location:** country: China; stateProvince: Yunnan; county: Jingdong; locality: Dujuanhu Lake; verbatimElevation: 2500 m; verbatimLatitude: 24° 32′ 22.76″ N; verbatimLongitude: 101° 1′ 6.85″ E; **Identification:** identifiedBy: H. W. Shen; Z. L. Luo; **Event:** year: 2023; month: 2; day: 24; habitat: Freshwater; **Record Level:** institutionID: KUN-HKAS; institutionCode: Herbarium of Kunming Institute of Botany, Academia Sinica (KUN-HKAS)

#### Description

**Figure 1.**
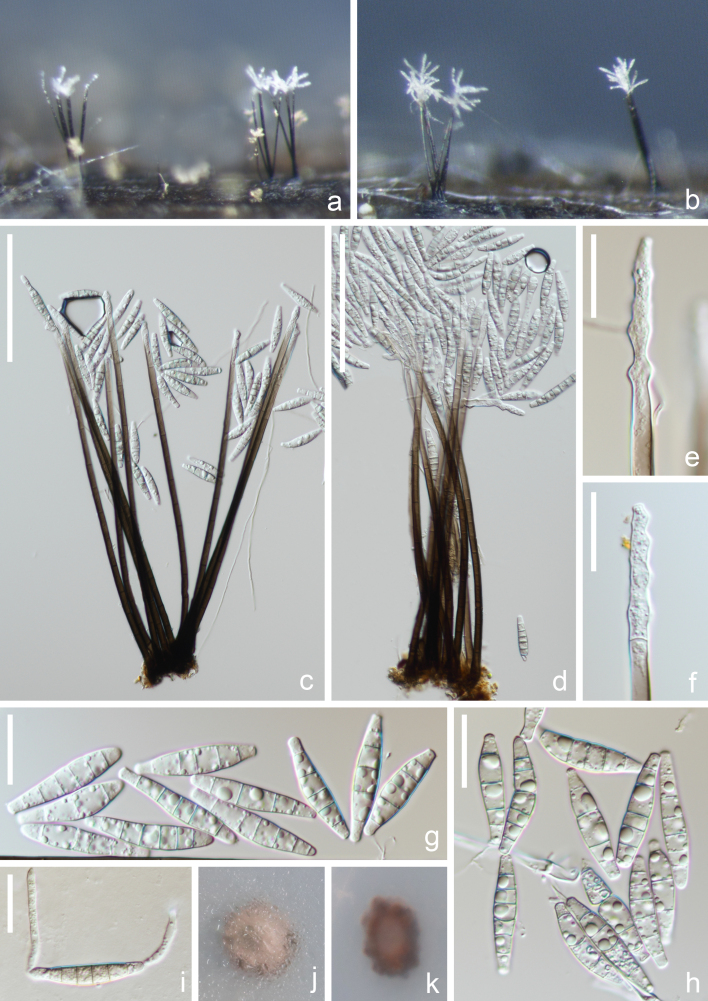
*Paramirandinaguttulata* (HKAS 131771, holotype) **a**, **b** Colony on natural substrate; **c**, **d** Conidiophores with conidia; **e**, **f** Conidiogenous cells; **g**, **h** Conidia; **i** Germinated conidium; **j**, **k** Culture on PDA medium. Scale bars: **c**, **d** = 100 μm, **e**–**i** = 20 μm.

*Saprobic* on submerged decaying wood in a freshwater lake. **Sexual morph**: Undetermined. **Asexual morph**: Hyphomycetous (Fig. 1). *Colonies* on natural substrates effuse, hairy, mostly in small groups, sometimes scattered, dark brown, with whitish conidial masses at the apex. *Mycelium* mostly immersed, composed of branched, septate, hyaline to brown, smooth-walled hyphae. *Conidiophores* (230–)264–310(–330) × 4.5–5(–6) µm (x̄ = 287 × 5 µm, n = 35), macronematous, mononematous, erect, gregarious, unbranched, cylindrical, straight to flexuous, septate, gradually tapering from the base to the apex, with the base transitioning from dark brown to hyaline towards the apex. *Conidiogenous cells* (31–)40–60(–70) × 4–5 µm (x̄ = 50 × 5 µm, n = 20), polyblastic, integrated, terminal, indeterminate, sympodial, cylindrical, pale brown to hyaline, smooth. *Conidia* (31–)33–38(–44) × 6–7.5 µm (x̄ = 35 × 7 µm, n = 40), acropleurogenous, solitary or in chains, fusiform, cymbiform, fusoid-clavate, straight or slightly curved, truncate at the base, obtuse at the apex, hyaline, 4–6-septate, smooth, guttulate, sometimes bearing a new conidium at the apex.

Culture characteristics: Conidia germinating on PDA medium and germ tubes produced from both ends of conidium within 12 h. Colonies on PDA medium reaching 1–1.5 cm diam. after one month at room temperature (around 25°C) in dark, circular, with dense, velvety, pale brown to brown mycelium from above, brown from below.

Material examined: CHINA, Yunnan Province, Pu’er City, Jingdong Yi Autonomous County, Dujuanhu Lake, 24°32′22.76″ N, 101°1′6.85″ E, on unknown decaying wood submerged in Dujuanhu Lake, H.W. Shen, 24 February 2023, L2204 (KUN-HKAS 131771, holotype), ex-type living culture DLUCC 2204.

#### Etymology

“*guttulata*” refers to guttulate conidia of the species.

#### Notes

*Paramirandinaguttulata* closely resemble *P.aquatica* and *P.cymbiformis*. However, they can be distinguished from each other by several key morphological characteristics. *Paramirandinaguttulata* can be distinguished from *P.aquatica* by its gregarious, longer conidiophores (264–310 × 4.5–5 µm *vs*. 138–200 × 4.5–8 μm) and slightly larger conidia (33–38 × 6–7.5 µm *vs*. 23–34 × 4–7.5 μm) (Liu et al. 2023). *Paramirandinaguttulata* differs from *P.cymbiformis* in having gregarious conidiophores and larger conidia (33–38 × 6–7.5 µm *vs*. 24–30 × 5–6.5 µm) (Liu et al. 2023). Comparisons of nucleotide base of ITS and LSU sequence data between *P.guttulata* and *P.aquatica* showed 7.5% (51/531 bp, including 11 gaps) and 1.6% (13/813 bp, without gaps) differences, respectively. The LSU sequences between *P.guttulata* and *P.cymbiformis* showed 1.6% (12/768 bp, without gaps) nucleotide base differences. Following the guidelines provided by Chethana et al. (2021), *P.guttulata* is introduced as a new species from the plateau lakes in Yunnan Province, China, based on its unique morphological characters and phylogenetic analysis.

## Analysis

**Figure 2.**
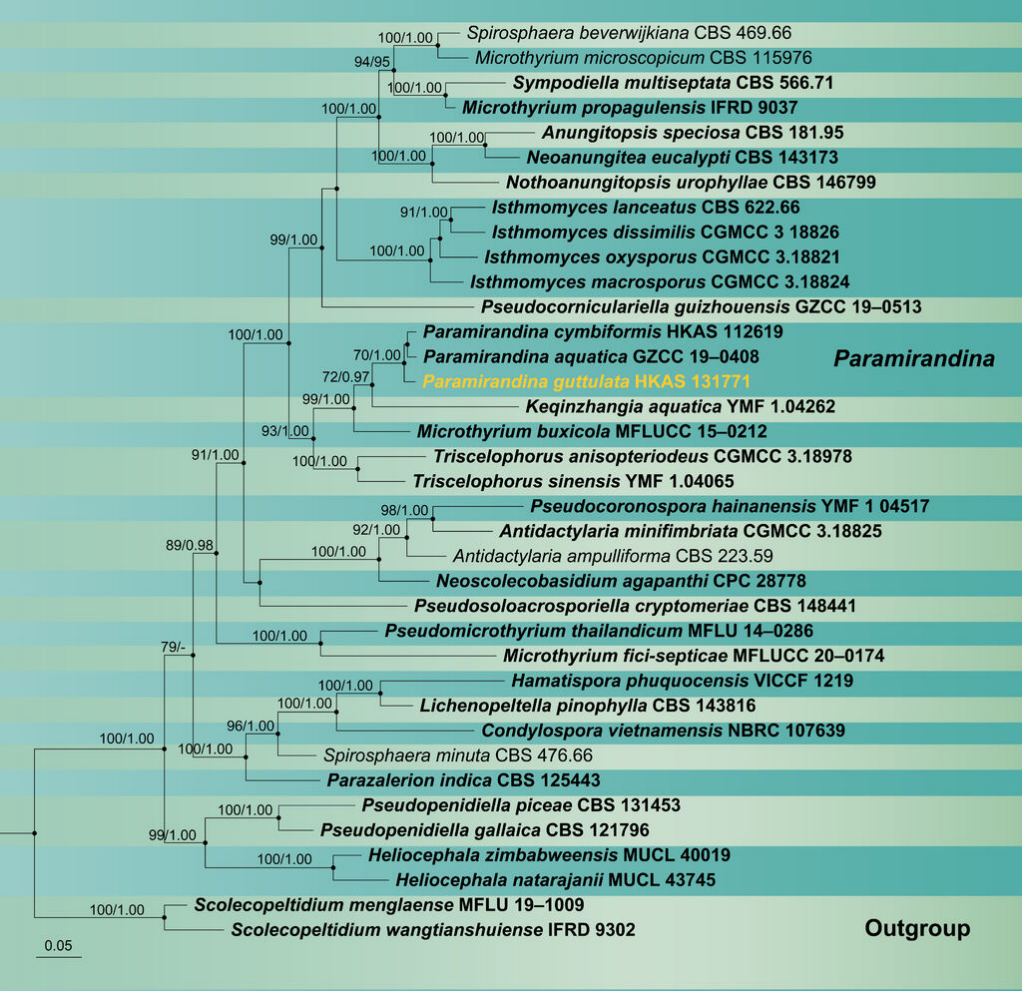
Maximum Likelihood (ML) tree is based on combined LSU and ITS sequence data. Bootstrap support values with a ML greater than 70% and Bayesian posterior probabilities (PP) greater than 0.95 are given above the nodes, shown as “ML/PP”. The tree is rooted to *Scolecopeltidiummenglaense* (MFLU 19–1009) and *S.wangtianshuiense* (IFRD 9302). New species are indicated in yellow and type strains are in bold.

Phylogenetic analysis was conducted on combined LSU and ITS sequence data of *Microthyriales* taxa. Thirty-seven strains are included in the phylogenetic analysis and the combined aligment comprise 1356 characters including gaps (860 characters for LSU, 496 characters for ITS). *Scolecopeltidiummenglaense* (MFLU 19–1009) and *S.wangtianshuiense* (IFRD 9302) were selected as outgroup taxa. Phylogenetic trees generated from Maximum Likelihood and Bayesian Inference analyses were similar in overall topologies (Fig. 2). Likelihood of the final tree is evaluated and optimised under GAMMA. The best RAxML tree with a final likelihood value of -17036.155151 is presented. The matrix contained 827 distinct alignment patterns, with 13.01% undetermined characters or gaps. Estimated base frequencies were as follows: A = 0.266021, C = 0.206060, G = 0.283009, T = 0.244910; substitution rates AC = 0.844979, AG = 2.331968, AT = 1.568434, CG = 0.748851, CT = 4.187264, GT = 1.000000, α = 0.326235, Tree-Length: 4.315793. Bayesian analyses generated 22002 trees (average standard deviation of split frequencies: 0.001160) from which 16502 were sampled after 25% of the trees were discarded as burn-in. The alignment contained a total of 827 unique site patterns. Bootstrap support values with a ML greater than 70% and Bayesian posterior probabilities (BYPP) greater than 0.95 are given above the nodes. Phylogenetic analysis showed that *Paramirandinaguttulata* clustered with the other two *Paramirandina* species and formed a basal lineage with 70% ML and 1.00 BYPP support (Fig. 2).

## Discussion

Microthyriaceae is phylogenetically poorly studied with limited molecular sequence data available in public databases (Wu et al. 2011b, Wu et al. 2014, Qiao et al. 2021, Zheng et al. 2022, Liu et al. 2023). Based on morphological characteristics and phylogenetic analysis, Microthyriaceae is currently considered the only family within Microthyriales, comprising a total of 18 accepted genera (Hongsanan et al. 2020, Qiao et al. 2021, Zheng et al. 2022, Liu et al. 2023). Species of Microthyriaceae are commonly found as foliar epiphytes or saprobes on dead leaves and stems (Wu et al. 2011b, Wu et al. 2014) and some hyphomycetes are reported on decaying wood and leaves submerged in freshwater habitats (Ingold 1943, Petersen 1962, Nawawi 1975, Qiao et al. 2021, Zheng et al. 2022, Liu et al. 2023). Notably, species of *Isthmomyces*, *Keqinzhangia*, *Paramirandina* and *Pseudocoronospora* have only been discovered in freshwater habitats (Qiao et al. 2021, Zheng et al. 2022, Liu et al. 2023).

*Paramirandina* was recently introduced by Liu et al. (2023) and comprises two species from lotic freshwater habitats on the Yunnan-Guizhou Plateau (Liu et al. 2023). In this study, we introduce the third species in this genus, also collected from freshwater habitats. The results of combined morphological and phylogenetic analysis demonstrated that the three *Paramirandina* species from freshwater habitats were consistently clustered within Microthyriaceae. Morphologically, the three species exhibited minimal variation, with only minor differences in the size of conidia and conidial cells. However, there is a significant difference in the size of the conidiophores which may serve as distinguishing features amongst species within this group. Furthermore, significant differences were found in their ITS and LSU molecular sequences, akin to several genera such as *Alternaria*, *Periconia*, *Pestalotiopsis* and *Torula* (Gu et al. 2022, Li et al. 2023, Su et al. 2023, Tian et al. 2023). In addition to ITS and LSU sequence data, there are notable nucleotide differences in the *tef*1-α and *rpb*2 sequences amongst *Paramirandina* species (Liu et al. 2023). However, due to the limited availability of *tef*1-α and *rpb*2 sequence data for species used in phylogenetic analysis, information from these markers may be insufficient for comprehensive analysis. Therefore, protein-coding gene fragments, such as *tef*1-α and *rpb*2, should be actively included in future studies. As more species are documented, these protein-coding sequences may prove valuable for species-level differentiation. The discovery of *Paramirandinaguttulata* not only augmented the species richness and distribution of this genus, but also enhanced the overall diversity of lignicolous freshwater fungi in a plateau lake in Yunnan Province, China.

## Supplementary Material

XML Treatment for Paramirandina guttulata

## Figures and Tables

**Table 1.** Taxa used in the phylogenetic analyses and their corresponding GenBank accession numbers. The ex-type strains are indicated using “^T^” after strain numbers; newly-generated sequences are indicated in bold. “–” stands for no sequence data in GenBank.

**Taxon**	**Voucher/Strain Number**	**GenBank Accession Number**	
		**LSU**	**ITS**
*Antidactylariaampulliforma*	CBS 223.59	MH869386	MH857845
*Antidactylariaminifimbriata*	CGMCC 3.18825 ^T^	MK577808	MK569506
*Anungitopsisspeciosa*	CBS 181.95 ^T^	EU035401	EU035401
*Condylosporavietnamensis*	NBRC 107639 ^T^	LC146725	LC146723
*Hamatisporaphuquocensis*	VICCF 1219 ^T^	LC064073	LC064074
*Heliocephalanatarajanii*	MUCL 43745 ^T^	HQ333480	HQ333480
*Heliocephalazimbabweensis*	MUCL 40019 ^T^	HQ333481	HQ333481
*Isthmomycesdissimilis*	CGMCC 3 18826 ^T^	MK577811	MF740794
*Isthmomyceslanceatus*	CBS 622.66 ^T^	MH870563	MH858897
*Isthmomycesmacrosporus*	CGMCC 3.18824 ^T^	MK577812	MF740796
*Isthmomycesoxysporus*	CGMCC 3.18821 ^T^	MK577810	MF740793
*Keqinzhangiaaquatica*	YMF 1.04262 ^T^	MK577809	MK569507
*Lichenopeltellapinophylla*	CBS 143816 ^T^	MG844152	–
*Microthyriumbuxicola*	MFLUCC 15-0212 ^T^	KT306551	–
*Microthyriumfici-septicae*	MFLUCC 20-0174 ^T^	MW063252	–
*Microthyriummicroscopicum*	CBS 115976	GU301846	–
*Microthyriumpropagulensis*	IFRD 9037 ^T^	KU948989	–
*Neoanungiteaeucalypti*	CBS 143173 ^T^	MG386031	MG386031
*Neoscolecobasidiumagapanthi*	CPC 28778 ^T^	NG_059748	NR_152546
*Nothoanungitopsisurophyllae*	CBS 146799 ^T^	MW883825	MW883433
*Paramirandinaaquatica*	GZCC 19-0408 ^T^	OQ025201	OQ025199
*Paramirandinacymbiformis*	HKAS 112619 ^T^	OQ025202	–
***Paramirandinaguttulata***	**HKAS 131771 ^T^**	**PQ345846**	**PQ345848**
*Parazalerionindica*	CBS 125443 ^T^	MH874977	MH863483
*Pseudocorniculariellaguizhouensis*	GZCC 19-0513 ^T^	OQ025203	OQ025200
*Pseudocoronosporahainanensis*	YMF 1.04517 ^T^	MK577807	MK569505
*Pseudomicrothyriumthailandicum*	MFLU 14-0286 ^T^	MT741680	–
*Pseudopenidiellagallaica*	CBS 121796 ^T^	LT984843	LT984842
*Pseudopenidiellapiceae*	CBS 131453 ^T^	JX069852	JX069868
*Pseudosoloacrosporiellacryptomeriae*	CBS 148441 ^T^	NG_081320	NR_175206
*Scolecopeltidiummenglaense*	MFLU 19-1009 ^T^	MW003710	MW003724
*Scolecopeltidiumwangtianshuiense*	IFRD 9302 ^T^	NG_067860	NR_166263
*Spirosphaerabeverwijkiana*	CBS 469.66	HQ696657	HQ696657
*Spirosphaeraminuta*	CBS 476.66	HQ696659	HQ696659
*Sympodiellamultiseptata*	CBS 566.71 ^T^	MH872028	MH860264
*Triscelophorusanisopteriodeus*	CGMCC 3.18978 ^T^	MK577818	MK569511
*Triscelophorussinensis*	YMF 1.04065 ^T^	MK577820	MK569513
